# Supplementary figures and images for: Integration of the metabolome and transcriptome reveals indigo biosynthesis in Phaius flavus flowers under freezing treatment
Source: PeerJ. 2022 Mar 14;10:e13106. doi: 10.7717/peerj.13106 (PMC8929171; doi:10.7717/peerj.13106)

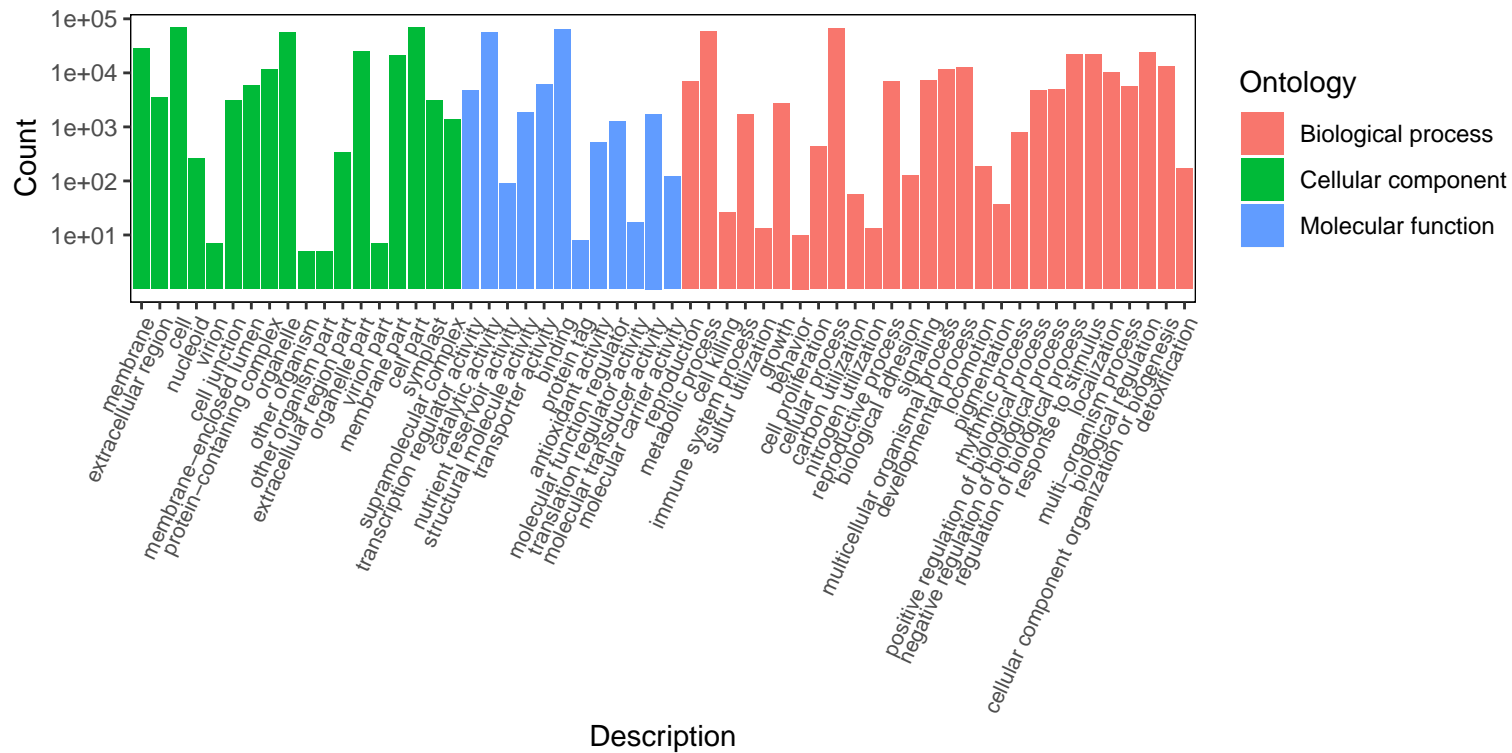

Supplement: Supplemental Information 1 [file peerj-10-13106-s001.pdf]
